# Asiatic acid attenuates methamphetamine-induced neuroinflammation and neurotoxicity through blocking of NF-kB/STAT3/ERK and mitochondria-mediated apoptosis pathway

**DOI:** 10.1186/s12974-017-1009-0

**Published:** 2017-12-11

**Authors:** Ji-Hyun Park, Young Ho Seo, Jung-Hee Jang, Chul-Ho Jeong, Sooyeun Lee, Byoungduck Park

**Affiliations:** 1 College of Pharmacy, Keimyung University, 1095 Dalgubeoldaero, Dalseo-Gu, Daegu, 42601 Republic of Korea; 2 Department of Pharmacology, School of Medicine, Keimyung University, 1095 Dalgubeoldaero, Dalseo-Gu, Daegu, 42601 Republic of Korea

**Keywords:** Methamphetamine, Asiatic acid, Neuroinflammation, Neurotoxicity, NF-κB, STAT3, ERK

## Abstract

**Background:**

Methamphetamine (METH) is a commonly abused drug that may result in neurotoxic effects. Recent studies have suggested that involvement of neuroinflammatory processes in brain dysfunction is induced by misuse of this drug. However, the mechanism underlying METH-induced inflammation and neurotoxicity in neurons is still unclear. In this study, we investigated whether asiatic acid (AA) effected METH-mediated neuroinflammation and neurotoxicity in dopaminergic neuronal cells. And we further determined whether the effect involved in the nuclear factor kappa-light-chain-enhancer of activated B cells (NF-κB) and signal transducer and activator of transcription (STAT)3 and extracellular signal-regulated kinase (ERK) pathway.

**Methods:**

We used the human dopaminergic neuroblastoma SH-SY5Y cell line, murine microglial BV2 cell line, and primary culture of rat embryo mesencephalic neurons. Pro-inflammatory cytokine production was monitored by ELISA and RT/real-time PCR. The cell cycle distribution and mitochondrial membrane integrity was analyzed by flow cytometry. We used immunoblotting, DNA-binding activity, and immunofluorescence staining to analyze the effect of AA on activation of the NF-κB, STAT3, MAPK-ERK, and apoptosis signaling pathways.

**Results:**

METH induced TNF receptor (TNFR) expression and led to morphological changes of cells. Additionally, this drug increased pro-inflammatory cytokine (TNFα and IL-6) expression. AA significantly suppressed METH-induced TNFR expression in concentration dependent. Increased secretion of TNFα and IL-6 was inhibited in METH-stimulated neuronal cells by AA administration. AA showed significant protection against METH-induced translocation of NF-κB/STAT3 and ERK phosphorylation. AA inhibited METH-induced proteolytic fragmentation of caspase-3 and PARP. The pro-apoptotic protein Bax was significantly decreased, while the anti-apoptotic protein Bcl-xL was increased by AA treatment in METH-stimulated cells. A similar protective effect of AA on mitochondrial membrane integrity was also confirmed by flow cytometry and immunofluorescence staining.

**Conclusions:**

Based on the literatures and our findings, AA is a promising candidate for an anti-neurotoxic agent, and it can potentially be used for the prevention and treatment of various neurological disorders.

**Electronic supplementary material:**

The online version of this article (10.1186/s12974-017-1009-0) contains supplementary material, which is available to authorized users.

## Background

Methamphetamine (METH) is a commonly abused drug that may result in neurotoxic effects, which contribute to neuronal damage and inflammation [1, 2]. It has been well-recognized that high doses of METH impair nigrostriatal dopaminergic systems in both rodents [3] and primates [4]. Although the pathogenesis of METH-induced dopaminergic neurotoxicity remains to be elucidated, this neurotoxicity may be, at least in part, related to oxidative stress [5, 6], inflammatory changes [7], and apoptosis [8]. METH may act upon neurons as a central processor of inflammation by releasing pro-inflammatory molecules [9]. These pro-inflammatory molecules may further activate downstream apoptotic signaling pathways in neurons, ultimately resulting in neuronal death and/or the activation of glial cells, which can further exacerbate neuroinflammation. In addition, the pathogenesis of dopaminergic neurotoxicity observed in Parkinson’s disease (PD) is similar to the neuroinflammatory and neurotoxic effect of METH [10].

Several studies have reported the neurotoxic mechanism of METH, including oxidative stress and apoptosis in neuronal cells [11–13]. METH causes induction of pro-inflammatory mediators such as inducible nitric oxide synthase (iNOS), interleukin (IL)-1β, IL-6, and tumor necrosis factor (TNF)-α [1, 14, 15]. Specifically, TNFα has been reported to be a potent stimulator of IL-6 production, whose pleiotropic action can be through the TNF receptor (TNFR) [9]. The activation of TNFR stimulates several signaling pathways that regulate cellular processes, ranging from cell proliferation and differentiation to cell death [16]. With respect to IL-6, its production seems to be regulated by several signaling cascades [9, 17], including TNF-α activation via the nuclear factor kappa-light-chain-enhancer of activated B cells (NF-κB) and signal transducer and activator of transcription (STAT) signaling pathways [14, 15, 18].

Increasing evidence has revealed that METH-induced neurodegeneration is associated with mitochondria-dependent apoptosis [19]. In addition, administration of METH in mice has been shown to cause an increase in pro-apoptotic proteins (BAX, BAD, and BID) but a decrease in anti-apoptotic Bcl-2-related proteins [11]. METH-induced alterations in these proteins have been suggested to form channels that result in mitochondrial membrane potential loss and allow cytochrome c release [20]. These observations propose that mitochondrial damage may contribute to METH-induced neurotoxicity. A direct cytotoxic role of METH has been found to be mediated by the mitogen-activated protein kinase (MAPK) pathway followed by the activation of caspases and the induction of apoptosis [21]. Although extensive research has focused on development of pharmacological agents that inhibit METH-induced neurotoxicity, FDA-approved pharmacotherapies for the treatment of negative effects of METH are still lacking [22]. Novel approaches aimed at overcoming the negative effects of METH are urgently needed in this field.

Asiatic acid (AA), a natural pentacyclic triterpene derived from the medicinal herb *Centella asiatica*, elicits multiple bioactivities, including antioxidant, anti-apoptotic, and anti-inflammatory properties [23–26]. A previous study showed that AA attenuates glutamate-induced cognitive deficits in mice and apoptosis in human neuroblastoma dopaminergic SH-SY5Y cells [27]. AA improves learning and memory in an animal model, which was correlated with an increase in hippocampal neurogenesis [28–30]. Although studies on the physiological function of AA, including anti-inflammatory and anti-apoptotic functions, were performed, the effectiveness of AA related to METH-induced neurotoxicity has not been evaluated. Therefore, in this study, we investigated the potential therapeutic effects of AA on the production of pro-inflammatory cytokines induced by METH. We also evaluated the molecular impact of AA on the signal transduction pathways involved in METH-induced neurotoxicity.

## Methods

### Cell cultures and reagents

A dopaminergic human neuroblastoma cell line SH-SY5Y (America Tissue Culture Collection, CRL-2266; ATCC, VA, USA) was cultured in a Dulbecco’s modified Eagle’s medium (DMEM) (Gibco, NY, USA) containing 10% fetal bovine serum (FBS; Gibco) and 1% Anti-Anti (Gibco). BV-2 murine microglia cells, a generous gift from Dr. Hoe (Korea Brain Research Institute, KBRI), were cultured in DMEM containing 10% FBS and 0.1% gentamicin (Gibco). Mesencephalic neuron cultures were prepared from the ventral mesencephalic tissues of embryonic day 13–14 rats, as described previously [31]. All experimental protocols were approved by the Institutional Animal Care and Use Committee of the Keimyung University (Daegu, South Korea; EXP-IRB number: KM_2016-007; the date of approval: 1 April 2016) in accordance with the criteria outlined in the Institutional Guidelines for Animal Research. Briefly, dissociated cells were seeded at 1 × 10^5^/well to poly-d-lysine- and laminin-coated 24-well plates. Cells were cultured in a Dulbecco’s modified Eagle’s medium/Ham’s F-12 medium (Gibco) containing ITS premix (Sigma-Aldrich, MO, USA) and 1% penicillin-streptomycin (Gibco). Cell cultures were maintained at 37 °C in a humidified atmosphere of 5% CO_2_.

Methamphetamine (METH) was purchased from the Ministry of Food and Drug Safety (Korea). Asiatic acid (AA) was purified and received from Dr. Ki Yong Lee, a professor of the College of Pharmacy, Korea University. Signal inhibitors were obtained from Sigma-Aldrich (MO, USA).

### Morphological examination

Morphological changes in cells were observed under an inverted phase-contrast microscope (Leica, Germany). The effect of AA on METH-induced neuroinflammation was observed for 24 h, and METH-induced neurotoxicity was observed for 12 h. The photographs were taken at ×200 or ×400 magnification using a digital camera.

### Cytotoxicity assay

Cells were plated in 96-well culture plates at 1 × 10^6^ cells/ml in culture medium and allowed to attach for 24 h. Media were then discarded and replaced with new medium containing various concentrations of METH and AA. The cells were cultured for an additional 24 h, and then, 3-(4, 5-dimethylthiazol-2-yl)-2,5-diphenylterazolium bromide (MTT, 5 mg/ml; Sigma-Aldrich) was added to each well (1/10 of medium volume), and the samples were incubated at 37 °C in a 5% CO_2_ incubator for 4 h. The formazan precipitate was dissolved in dimethyl sulfoxide (DMSO), and absorbance was measured at 540 nm using a microplate reader (Bio-Rad Laboratories, CA, USA).

### Treatment kinase inhibitors and siRNA transfection

Cells were pretreated with various inhibitors (Sigma-Aldrich) such as NF-κB-specific inhibitor: Bay11-7085 (20 μM), STAT3-specific inhibitor: S3I-201 (20 μM), and ERK1/2-specific inhibitor: PD98059 (20 μM). After 1 h, the cells were treated and co-cultured with METH for 24 h. Cells were transfected with control siRNA (Cat no: sc-37007, Santa Cruz, CA, USA) and ERK-MAPK siRNA (Cat no: 6560, Cell Signaling Technology, MA, USA) using Lipofectamine RNAiMAX transfection reagent (Invitrogen) according to the manufacturer’s instruction.

### Enzyme-linked immunosorbent assay (ELISA)

The culture medium of the cells was harvested, and cytokine production (TNFα and IL-6) in the supernatant was measured with a solid-phase sandwich enzyme-linked immunosorbent assay (ELISA) using a Quantikine TNF-α and IL-6 kit (R&D systems, MN, USA) according to the manufacturer’s instructions.

### Immunoblot analysis

Cytosolic and nuclei protein fractions were obtained as described [32]. The protein concentration was determined with a Bio-Rad Bradford kit (Bio-Rad Laboratories, CA, USA). The samples were boiled for 5 min, and equal volumes were loaded on a sodium dodecyl sulfate-polyacrylamide gel electrophoresis (SDS-PAGE). The resolved proteins were transferred onto a nitrocellulose membrane and probed with anti-TNFR, anti-p-NF-κB p65, anti-NF-κB p65, anti-p-STAT3, anti-STAT3, anti-p-JAK2, anti-JAK2, anti-p-ERK1/2, anti-ERK1/2, anti-p-JNK1/2, anti-JNK1/2, anti-p-p38, anti-p38, anti-TH, anti-lamin B, and anti-β-actin (all from Cell Signaling Technology) followed by a secondary antibody conjugated to horseradish peroxidase and detected with enhanced chemiluminescence reagents (Amersham Bioscience, UK). The luminescent signals were analyzed using an ImageQuant LAS 4000 Scanner of GE Healthcare (Piscataway, NJ, USA).

### Reverse-transcription and real-time PCR

Total RNA was extracted from neuronal cells with TRIzol reagent (Invitrogen Co, Grand Island, NT, USA) according to the manufacturer’s instructions. First-strand cDNA was synthesized with oligo-d(T) primer and M-MLV reverse transcriptase (Promega, Madison, WI, USA). Real-time PCR was performed in a CFX 96 Touch™ detection system (Bio-Rad Laboratories) using SYBR Green PCR Master Mix (Bio-Rad Laboratories). Each measurement was repeated at least in triplicate, and the relative quantity of target mRNA was determined using the comparative threshold (C_t_) method by normalizing target mRNA C_t_ values to those for β-actin (∆C_t_). Aliquots of cDNA were used for PCR using primer sets specific to TNF-α, IL-6, and β-actin as a control. Used primers are as follows: for TNF-α, 5′-TCT CGA ACC CCG AGT GAC AA-3′ (sense) and 5′-TGA AGA GGA CCT GGG AGT AG-3′ (antisense); for IL-6, 5′-CAC AGA CAG CCA CTC ACC TC-3′ (sense) and 5′-TTT TCT GCC AGT GCC TCT TT-3′ (antisense); and for β-actin, 5′-CTT CCT GGG CAT GGA GTC CT-3′ (sense) and 5′-GGA GCA ATG ATC TTG ATC TT-3′ (antisense).

### Electrophoretic mobility shift analysis (EMSA) and supershift assay

As described in our previous studies, the DIG Gel Shift Kit (Roche, Mannheim, Germany) was used to detect NF-κB-p65- and STAT3-binding activity, with the instructions of the manufacturer [32, 33]. The binding activity of NF-κB-p65 and STAT3 in the METH-induced cells was confirmed by EMSA or supershift assay with a DIG-labeled oligonucleotide (NF-κB: 5′-AGT TGA GGG GAC TTT CCC AGG C-3′, STAT3: 5′-CTT CAT TTC CCG TAA ATC CCT AAA GCT-3′) and NF-κB-p65 and STAT3 antibody (Cell Signaling Technology). The NF-κB-p65-oligo, NF-κB-p65-Ab, STAT3-oligo, and STAT3-Ab complexes were separated by electrophoresis on 6% non-denaturing polyacrylamide gels. After electrophoresis, the gels were transferred to nylon membranes and detected by chemiluminescence. The luminescent signals were analyzed on an ImageQuant LAS 4000 Scanner (GE Healthcare).

### Immunofluorescence staining

Cells were grown on chamber slides and coating cover slides and fixed with 3.7% paraformaldehyde in PBS. The membrane was permeabilized by treating cells for 2 min with 0.1% Triton X-100 in PBS. The cells were then placed in blocking solution (5% FBS in PBS) at room temperature. Cells were incubated with primary antibodies for 1 h at room temperature. After washing, they were incubated with the Alexa Flour 488 (excitation/emission = 495/519 nm, green, Invitrogen, CA, USA) and Alexa Flour 594 (excitation/emission = 590/617 nm, red, Invitrogen, CA, USA) for 30 min at room temperature. Cells were counterstained with Hoechst 33342 (excitation/emission = 330–380 nm/460 nm, ImmunoChemistry, MN, USA). Slides were mounted using ProLong® Gold antifade reagent (Molecular Probes® by Life Technologies™, CA, USA). Primary antibodies utilized are the following: anti-pNF-κB, anti-pSTAT3, and anti-tyrosine hydroxylase (TH) (from Cell Signaling Technology). Immunolabeling was examined using an Eclipse Ti-U and confocal microscope (Nikon, Tokyo, Japan).

### Annexin V/propidium iodide (PI) staining

Apoptotic cells were differentiated from viable or necrotic cells using a combined staining of annexin V-FITC and PI (Invitrogen). To investigate the neurotoxic effect of AA on METH, cells were pretreated with 20 μM AA for 1 h and then exposed to 1.5 mM METH for 12 h. After the cells were trypsinized, they were washed in PBS and resuspended in annexin-binding buffer. The cell suspension (1 × 10^5^ cells/ml) was incubated with FITC-conjugated anti-annexin V and PI for 15 min in the dark. Cells were counted using a BD FACSVerse™ (BD Biosciences, NJ, USA) and analyzed by Flowing 2.5 version software. A total number of 10,000 events were recorded for each sample. The fluorescence spectrum of annexin V and PI were detected using a 527/32-nm and a 586/42-nm filter, respectively.

### JC-1 mitochondrial transmembrane potential assay

To measure the mitochondrial transmembrane potential, JC-1 dye (Sigma-Aldrich), a sensitive fluorescent probe, was used. Fluorescence microscopy with a 488-nm filter was used for the excitation of JC-1. Emission filters of 535 and 595 nm were used to quantify the population of mitochondria with green (JC-1 monomers) and red (JC-1 aggregates) fluorescence, respectively. Immunolabeling was examined by an Eclipse Ti-U microscope (Nikon) and BD FACS Verse flow cytometer.

### Statistical analysis

All values are expressed as mean ± standard error of the mean (SEM). All data analysis was performed with the GraphPad Prism 5 (GraphPad Software, Inc., San Diego, CA) using either a one-way ANOVA with Tukey’s post hoc test for multiple comparisons, with *p* < 0.05 defined as significant. For the quantification of immunofluorescence, staining results are expressed as percentages of total cells stained with Hoechst 33342 per each field (*n* = number of fields). For all data, *n* corresponds to the number of independent experiments.

## Results

### Effects of AA on METH-induced production of pro-inflammatory cytokines

To determine cytotoxicity, dopaminergic SH-SY5Y cells (human neuroblastoma) treated with different concentrations of METH (0.5 to 5 mM) and AA (1 to 30 μM) for 24 h were analyzed by an established MTT assay. The treatment of SH-SY5Y cells with AA concentrations ranging from 1 to 20 μM showed mild growth inhibitory activity with a 10% decrease in cell viability at 20 μM but exhibited some toxicity at 30 μM (45%; Additional file 1: Figure S1a). The next experiment was performed to observe the viability and TNFα secretion of SH-SY5Y cells treated with METH for 24 h. Viability of METH-stimulated SH-SY5Y cells was decreased by about 30% as compared to that of control cells at 1 mM (Additional file 1: Figure S1b—upper). TNFα secretion was increased at 1 mM METH, and this was maintained similarly from 1.5 to 5 mM (Additional file 1: Figure S1b—lower). AA significantly increased the viability of 1 mM METH-stimulated SH-SY5Y cells in a concentration-dependent manner compared to that of cells treated with 1 mM METH alone (Additional file 1: Figure S1c). We also confirmed these results at the cell morphology level (Additional file 1: Figure S1d). SH-SY5Y cells showed healthy morphology with full cell bodies and extending neurites. After exposed to 1 mM METH, cells were sparsely distributed and displayed growth inhibition and development of short neurites with few branches. However, 20 μM AA significantly inhibited the cell damage of 1 mM METH-stimulated SH-SY5Y cells. This result is consistent with changes of cell viability. Based on these results, the optimal AA concentration for subsequent experiments was chosen as 20 μM for 1 mM METH-stimulated SH-SY5Y cells.

**Fig. 1:**
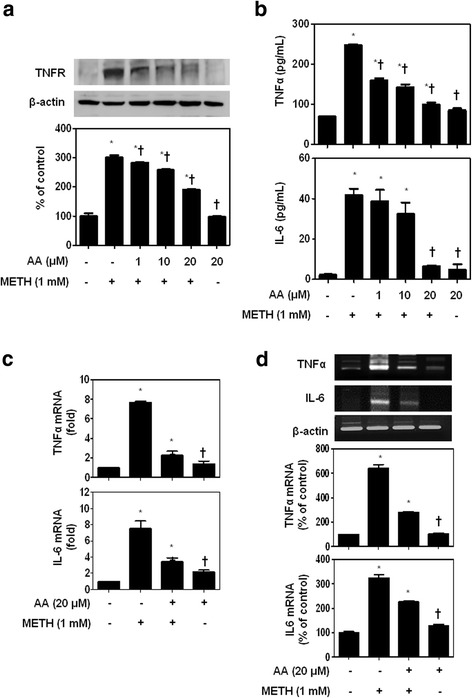
AA inhibits METH-induced TNF-alpha and IL-6 production and mRNA expression levels. SH-SY5Y cells were incubated in the presence or absence of AA (1, 10, and 20 μM) for 1 h and then treated with 1 mM METH for 24 h. **a** TNFR overexpression was significantly inhibited in METH-stimulated SH-SY5Y cells by AA administration. AA strongly suppressed METH-induced TNFα and IL-6 production both in extracellular (**b**) and mRNA levels (**c**, **d**). β-actin was used to confirm equal sample loading. The data are representative of three independent experiments and quantified as mean values ± SEM (*n* = 4 to 9). Tukey’s multiple comparison test, *p < 0.05 compared to normal control, ^†^ *p* < 0.05 compared to METH treatment

METH leads to rapid upregulation of pro-inflammatory cytokines such as TNFα and IL-6 through TNFR [9]. To determine whether AA can regulate METH-induced TNFR expression, SH-SY5Y cells were incubated in the presence or absence of AA for 1 h and then treated with METH for 24 h. AA significantly suppressed METH-induced TNFR expression in a concentration dependent (Fig. 1a). We next analyzed the effect of AA on METH-induced secretion of TNFα and IL-6 by ELISA. Increased TNFα and IL-6 secretion was significantly inhibited in METH-stimulated SH-SY5Y cells by AA administration (Fig. 1b). We also confirmed these results at the mRNA level. Consistent with the ELISA results, AA strongly suppressed METH-induced TNFα and IL-6 mRNA expression (Fig. 1c, d). Taken together, our results indicate that AA inhibits METH-induced expression of TNFα and IL-6 at the level of mRNA, which resulted in inhibition of the pro-inflammatory cytokine production in dopaminergic SH-SY5Y cells.

### AA inhibits pro-inflammatory cytokine secretion through suppression of NF-κB, STAT3, and ERK pathway

**Fig. 2:**
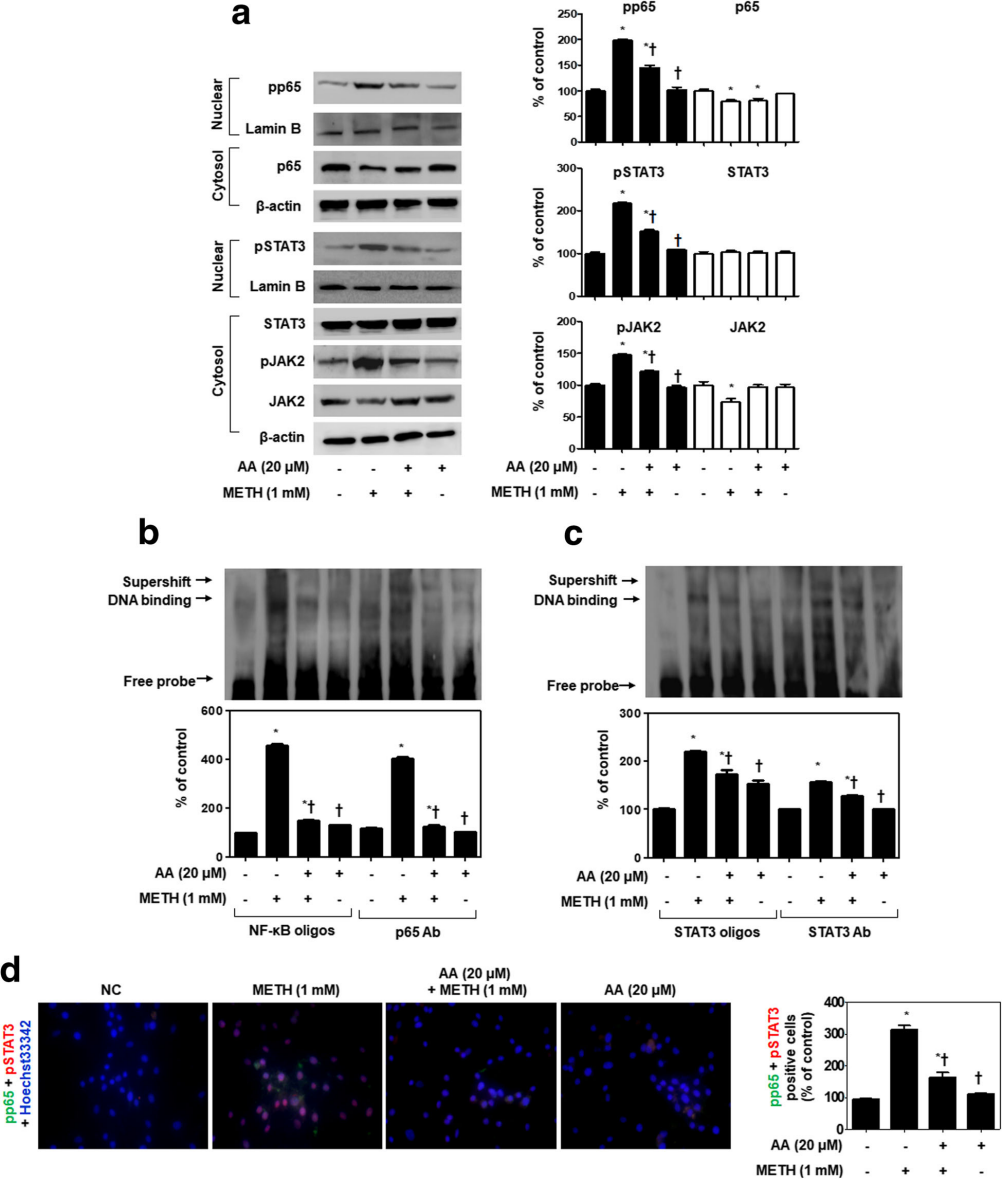
AA suppresses the translocation of NF-κB and STAT3 in METH-stimulated SH-SY5Y cells. **a** Immunoblot analysis shows the effects of 20 μM AA on the inhibition of translocation of NF-κB/STAT3 and phosphorylation of JAK2 (an upstream activator of STAT3) in 1 mM METH-stimulated SH-SY5Y cells. β-actin and lamin B were used to confirm equal sample loading. DNA- and Ab-binding activity of NF-κB (**b**) and STAT3 (**c**) in nuclear extracts was measured by EMSA and supershift assay. **d** Immunofluorescence double staining for p-NF-κB p65 (green) and p-STAT3 (red) localization. Cells were counterstained with Hoechst 33342 (blue). Magnifications ×200. Immunoblotting, EMSA, and supershift assay were quantified by densitometric analysis. The data are representative of three independent experiments and quantified as mean values ± SEM (*n* = 3 to 4). Tukey’s multiple comparison test, *p < 0.05 compared to normal control, ^†^ *p* < 0.05 compared to METH treatment

NF-κB and STAT3 activation is known to be a regulatory mechanism for TNFα and IL-6 [34]. Therefore, we examined the translocation of NF-κB and STAT3 in response to METH-induced neuroinflammation to SH-SY5Y cells (Fig. 2a). SH-SY5Y cells were pretreated with 20 μM AA for 1 h and then stimulated with 1 mM METH for 24 h. Along with phosphorylation, NF-κB-p65 and STAT3 were translocated from the cytoplasm to the nucleus after METH stimulation, but this was effectively inhibited by AA. Moreover, AA strongly reduced METH-induced phosphorylation of JAK2. We further evaluated the effects of AA on METH-induced NF-κB and STAT3 DNA-binding activity (Fig. 2b, c). The nuclear extracts of SH-SY5Y cells, which were incubated in the presence or absence of AA, were analyzed using DIG-labeled oligonucleotides corresponding to the NF-κB and STAT3 sites. Formation of NF-κB-DNA, NF-κB-Ab, STAT3-DNA, and STAT3-Ab complexes was prominent in nuclear extracts from METH-stimulated SH-SY5Y cells. However, formation of these complexes was significantly suppressed in METH-stimulated SH-SY5Y cells when these cells were treated with AA. We performed immunofluorescence staining to confirm whether treatment with AA could inhibit nuclear translocation of NF-κB and STAT3 (Fig. 2d, Additional file 2: Figure S2). The translocation of NF-κB and STAT3 was observed at the same position as the staining nucleus in METH-stimulated SH-SY5Y cells. These expressions were effectively inhibited by 20 μM AA. The results were entirely consistent with our earlier data.

**Fig. 3:**
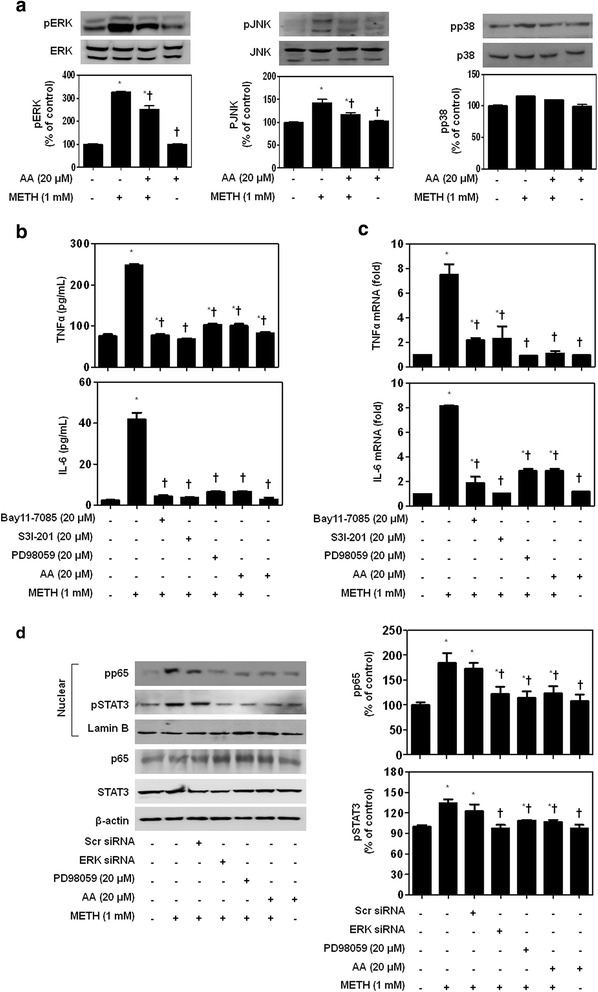
AA inhibits pro-inflammatory cytokine secretion through suppression of NF-κB, STAT3, and ERK pathway in METH-stimulated SH-SY5Y cells. SH-SY5Y cells were pretreated with 20 μM AA or various inhibitors for 1 h and then stimulated with 1 mM METH for 24 h. **a** Phosphorylation of ERK was strongly inhibited in METH-stimulated SH-SY5Y cells by AA administration. Inhibitors of NF-κB (Bay11-7085), STAT3 (S3I-201), ERK (PD98059), or AA strongly suppressed METH-induced TNFα and IL-6 production both in extracellular (**b**) and mRNA levels (**c**, **d**). METH-induced translocation of p65 and STAT3 was inhibited when the ERK pathway of these cells were downregulated. β-actin and lamin B were used to confirm equal sample loading. Immunoblotting was quantified by densitometric analysis. The data are representative of three independent experiments and quantified as mean values ± SEM (*n* = 4 to 6). Tukey’s multiple comparison test, *p < 0.05 compared to normal control, ^†^ *p* < 0.05 compared to METH treatment

The MAPK signaling pathway, ERK-JNK-p38 MAPKs, is involved in the regulation of pro-inflammatory cytokines [35]. Previous reports have revealed that METH administration can increase the expression of ERK and JNK in the brain [7, 21]. Accordingly, we investigated whether 20 μM influences 1 mM METH-induced pro-inflammatory cytokine expression via regulation of the ERK-JNK-p38 signaling pathway (Fig. 3a). Phosphorylation of ERK-JNK-p38 was significantly induced in METH-stimulated SH-SY5Y cells. AA inhibited METH-induced phosphorylation of ERK and JNK. However, phosphorylation of p38 was not affected. Interestingly, AA strongly inhibited METH-induced phosphorylation of ERK.

To confirm our finding that AA inhibits METH-induced pro-inflammatory cytokine expression through NF-κB, STAT3, and MAPK-ERK signaling, we performed the following experiment after treatment of cells with inhibitors of NF-κB (Bay11-7085), STAT3 (S3I-201), and ERK (PD98059). Consistent with the findings described above, Bay11-7085, S3I-201, PD98059, and AA significantly decreased METH-induced TNFα and IL-6 secretion and reduced these cytokines at the mRNA level (Fig. 3b, c).

To further investigate the role of ERK in METH-induced translocation of p65 and STAT3, cells were transfected with ERK siRNA or treated with the ERK inhibitor (PD98059). As shown in Fig. 3d, METH-induced translocation of p65 and STAT3 was inhibited when the ERK pathway of these cells were downregulated. Together, these data suggest that AA inhibits translocation of NF-κB/STAT3 and suppresses ERK phosphorylation, ultimately leading to inhibition of METH-induced pro-inflammatory cytokine production.

### AA inhibits the NF-κB, STAT3, and MAPK-ERK signaling pathway in METH-stimulated BV2 cells

**Fig. 4:**
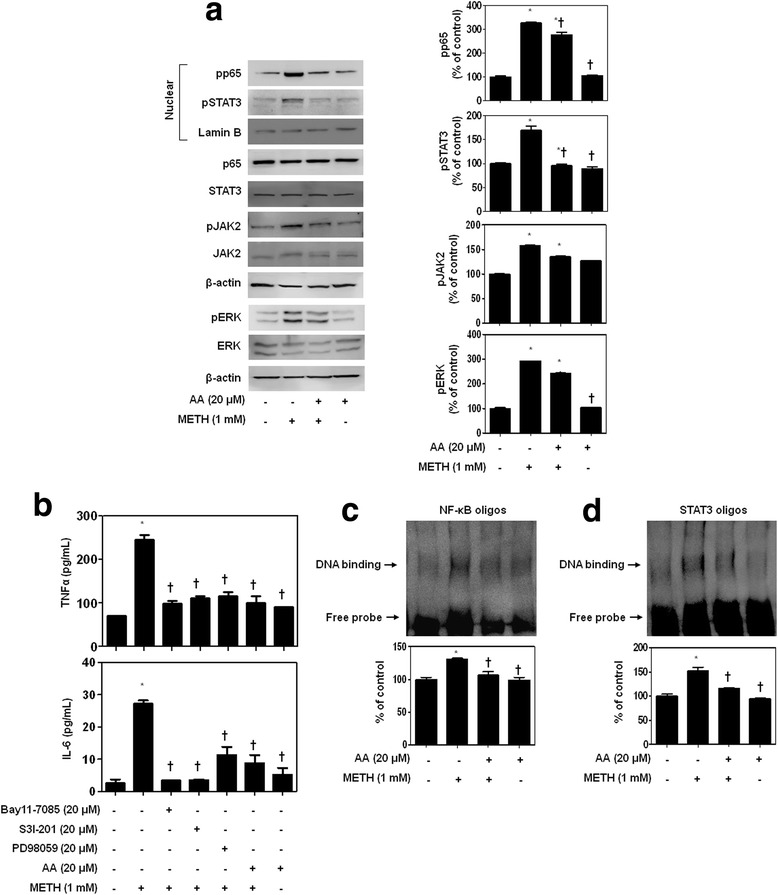
AA suppresses METH-stimulated TNFα and IL-6 production through inhibition of NF-κB/STAT3 and MAPK-ERK signaling pathways in microglia BV2 cells. BV2 cells were pretreated with 20 μM AA or various inhibitors for 1 h and then stimulated with 1 mM METH for 24 h. **a** 20 μM AA suppresses 1 mM METH-induced translocation of NF-κB/STAT3 and phosphorylation of JAK2/ERK in BV2 cells. β-actin and lamin B were used to confirm equal sample loading. **b** Inhibitors of NF-κB (20 μM Bay11-7085), STAT3 (20 μM S3I-201), ERK (20 μM PD98059), or 20 μM AA strongly suppressed METH-induced TNFα and IL-6 production in extracellular levels. DNA-binding activity of NF-κB (**c**) and STAT3 (**d**) in nuclear extracts was measured by EMSA. Immunoblotting and EMSA were quantified by densitometric analysis. The data are representative of three independent experiments and quantified as mean values ± SEM (*n =* 4 to 6). Tukey’s multiple comparison test, *p < 0.05 compared to normal control, ^†^ *p* < 0.05 compared to METH treatment

Microglia activation and the release of various inflammatory cytokines are largely related to neurological diseases [36]. Accordingly, we explored whether AA influences pro-inflammatory cytokine production via regulation of NF-κB, STAT3, and MAPK-ERK signaling pathways in microglia BV2 cells. BV2 cells were pretreated with 20 μM AA for 1 h and then stimulated with 1 mM METH for 24 h. In agreement with the results in Figs. 2 and 3, 1 mM METH strongly induced translocation of p65 and pSTAT3 in BV2 cells (Fig. 4a). In addition, phosphorylation of JAK2, an upstream activator of STAT3, and ERK was induced by METH in BV2 cells. Twenty micromolar of AA inhibited 1 mM METH-induced translocation of NF-κB/STAT3 and phosphorylation of JAK2/ERK in BV2 cells.

It was further investigated whether AA affects METH-induced pro-inflammatory cytokine secretion through NF-κB, STAT3, and MAPK-ERK signaling. BV2 cells were pretreated with 20 μM AA or inhibitors (20 μM Bay11-7085, 20 μM S3I-201, 20 μM PD98059) for 1 h and then stimulated with 1 mM METH for 24 h. When we examined the effects of AA on METH-induced pro-inflammatory cytokine expression following treatment with Bay11-7085, S3I-201, PD98059, and AA, these all significantly decreased METH-induced TNFα and IL-6 secretion (Fig. 4b). Subsequently, we examined whether 20 μM AA inhibits 1 mM METH-induced NF-κB and STAT3 DNA-binding activity (Fig. 4c, d). Increased formation of NF-κB-DNA and STAT3-DNA complexes was suppressed in 1 mM METH-stimulated BV2 cells with 20 μM AA treatment. Together, these results indicate that AA strongly inhibits METH-induced pro-inflammatory cytokine expression in microglia cells.

### AA inhibits the neuroinflammatory pathway in METH-stimulated tyrosine hydroxylase (TH)-positive neurons

**Fig. 5:**
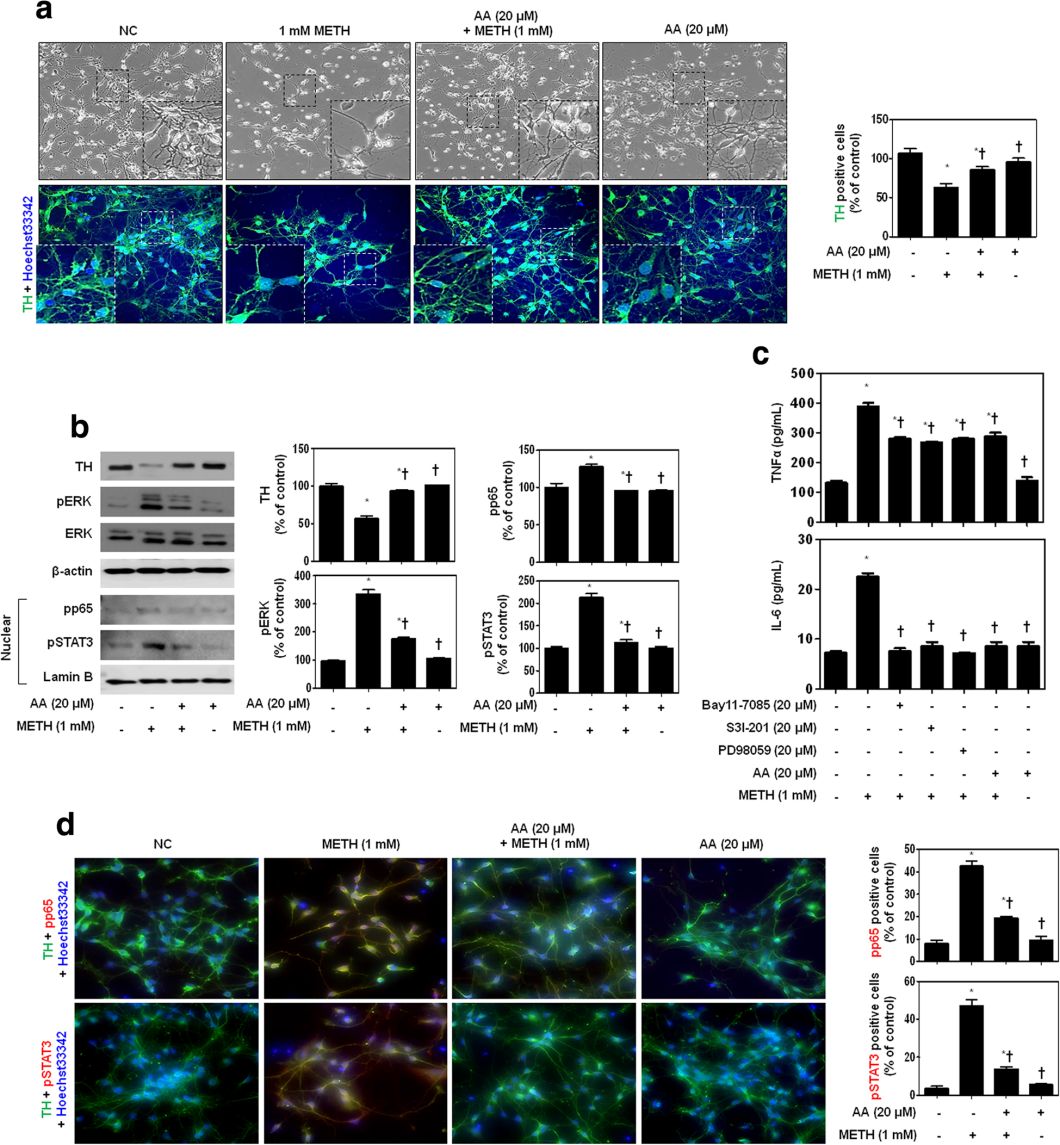
Protective effects of AA on METH-stimulated mesencephalic neurons. **a** Along with the presence or absence of AA, the morphological changes (upper) and TH expression (lower) in dopaminergic neurons after exposure to METH. Immunofluorescence staining for TH (green) localization. Cells were counterstained with Hoechst 33342 (blue). Magnifications ×400. **b** AA strongly reverses TH expression and inhibits phosphorylation of ERK and translocation of NF-κB/STAT3. β-actin and lamin B were used to confirm equal sample loading. Immunoblotting was quantified by densitometric analysis. **c** Inhibitors of NF-κB (Bay11-7085), STAT3 (S3I-201), ERK (PD98059), or AA significantly suppresses TNFα and IL-6 secretion in METH-stimulated mesencephalic neurons. **d** Immunofluorescence double staining for TH (green) and p-NF-κB p65/p-STAT3 (red) localization. Cells were counterstained with Hoechst 33342 (blue). Magnifications ×200. The data are representative of three independent experiments and quantified as mean values ± SEM (*n* = 4 to 6). Tukey’s multiple comparison test, *p < 0.05 compared to normal control, ^†^ *p* < 0.05 compared to METH treatment

Primary neuronal cells were obtained from the ventral mesencephalic tissues of embryonic day 13–14 rats. Cultures of mesencephalic neurons are widely used as a source of dopaminergic neurons for the study of mechanisms implicated in dopaminergic cell death and for the evaluation of potential dopaminergic neuroprotective agents, including neurotrophic factors [37]. Therefore, we next analyzed the effect of AA on METH-induced secretion of pro-inflammatory cytokines and the translocation of NF-κB and STAT3 in mesencephalic neurons. These neurons were pretreated with AA for 1 h and then stimulated with METH for 24 h. In the presence or absence of AA, the morphological changes in dopaminergic neurons after exposure to METH were observed under an inverted microscope (Fig. 5a—upper). The normal control cells grew well showing obvious neurites, and the cells treated with AA alone did not show any difference in cell growth when compared with normal cells. When TH-positive neurons were exposed to METH, neurites were reduced and cell debris was increased; however, they were recovered with AA co-treatment. These results show that AA exerts a protective effect against cell damage induced by METH. Immunofluorescence analysis with an antibody against TH revealed that mesencephalic neuronal cells were healthy TH-positive neurons with extensive neurites (Fig. 5a—lower). However, treatment of neurons with METH for 24 h reduced the number of TH-positive cells. When the cells were treated with AA alone, the number and morphology of the TH-positive neurons were not changed. The addition of AA to METH-treated cells seemed to protect against the loss of TH-positive neurons. We also confirmed this result at the protein level. Consistent with immunofluorescence staining, 1 mM METH strongly induced TH reduction in mesencephalic neurons. This reduction was strongly reversed by AA in METH-stimulated mesencephalic neurons (Fig. 5b). In addition, AA showed significant inhibition against METH-induced phosphorylation of ERK and translocation of NF-κB/STAT3 in mesencephalic neurons.

Next, we further investigated whether Bay11-7085, S3I-201, and AA play a role in regulating METH-induced pro-inflammatory cytokine production. Mesencephalic neurons were pretreated with 20 μM AA or inhibitors (20 μM Bay11-7085, 20 μM S3I-201, 20 μM PD98059) for 1 h and then stimulated with 1 mM METH for 24 h. As shown in Fig. 5c, secretion of TNFα by METH was slightly higher in mesencephalic neurons than the SH-SY5Y and BV2 cells. Bay11-7085, S3I-201, PD98059, and AA significantly decreased secretion in METH-stimulated mesencephalic neurons. Accordingly, METH-induced IL-6 secretion was also decreased by AA or inhibitors. To confirm our results, we conducted immunofluorescence staining using pNF-κB and pSTAT3 antibodies (Fig. 5d, Additional file 3: Figure S3 and Additional file 4: Figure S4). Consistent with the findings presented above, localization of pNF-κB and pSTAT3 to the nucleus was suppressed in METH-stimulated mesencephalic neurons by AA.

### AA ameliorates METH-induced neurotoxicity in dopaminergic SH-SY5Y cells

METH-induced apoptosis in neuronal cells is associated with the activation of NF-κB, STAT3, and MAPK-ERK signaling [15, 18, 38]. We first analyzed the caspase-3 (17 kDa) and PARP (89 kDa) expression to understand the neurotoxic effects of METH on the growth of dopaminergic SH-SY5Y cells (Additional file 5: Figure S5). We treated SH-SY5Y cells with METH (0.5, 1, 1.5, 2, and 5 mM) for 12 h and then conducted immunoblotting and examined cell morphology. METH-stimulated SH-SY5Y cells showed increased cleavage of caspase-3 (17 kDa) and PARP (89 kDa) in a concentration dependent. Full length of PARP was slightly detected from 2 mM of METH treated cells. So 1.5 mM of METH was chosen to confirm the inhibitory effect on METH neurotoxicity.

**Fig. 6:**
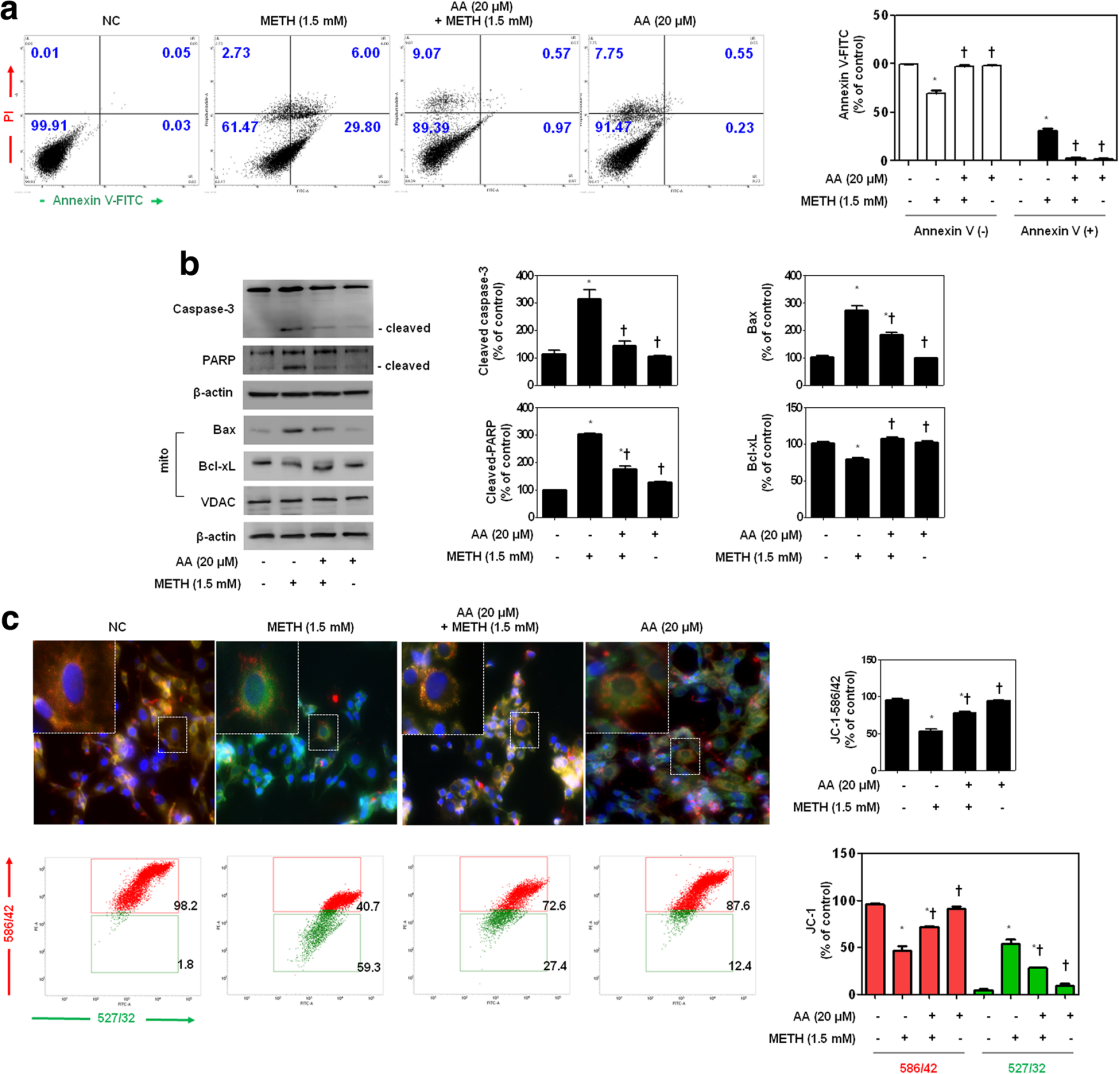
AA protects METH-induced apoptosis in SH-SY5Y cells. **a** Annexin V/PI staining by flow cytometry. AA inhibits cleavages of caspase-3 and PARP (**b**) and regulates apoptotic proteins (**c**). VDAC was used as mitochondrial loading control, and β-actin was used to confirm equal sample loading. Immunoblotting was quantified by densitometric analysis. SH-SY5Y cells were evaluated by morphological criteria after JC-1 mitochondria staining (red and green) by using fluorescent microscope (upper) and flow cytometry (lower). Magnifications ×200. The data are representative of three independent experiments and quantified as mean values ± SEM (*n* = 3 to 9). Tukey’s multiple comparison test, *p < 0.05 compared to normal control, ^†^ *p* < 0.05 compared to METH treatment

Next, to determine the effect of AA, the distribution of apoptosis cells by METH conducted flow cytometry with annexin V/PI staining. Flow cytometric analyses showed that 35.8% of the METH-stimulated SH-SY5Y cells stained positively for annexin V. However, only 1.54% cells treated with AA prior to METH stained positively for annexin V. These results suggested that AA increases cell viability and inhibits apoptosis of METH-stimulated SH-SY5Y cells (Fig. 6a). METH-stimulated SH-SY5Y cells showed increased cleavage of caspase-3 (17 kDa) and PARP (89 kDa). The addition of AA inhibited METH-induced proteolytic fragmentation of caspase-3 and PARP (Fig. 6b).

To evaluate the effects of METH on the production of apoptotic proteins related to mitochondria, we assessed Bcl-2 family activation in METH-treated SH-SY5Y cells by immunoblot analysis. As shown in Fig. 6c, the pro-apoptotic protein Bax was significantly decreased, while the anti-apoptotic protein Bcl-xL was increased by AA treatment. Next, we used JC-1 staining to measure the mitochondrial membrane potential to examine whether mitochondrial membrane integrity is affected by AA. AA clearly recovered the number of mitochondria, which was observed as decreased membrane potential in METH-stimulated SH-SY5Y cells (Fig. 6d). A similar protective effect of AA on mitochondrial membrane integrity was also confirmed by flow cytometric analyses. When SH-SY5Y cells were treated with METH, green fluorescence increased from 1.8% in the normal control to 59.3%. The percentage of cells that fluoresced red decreased to 40.7% after METH treatment. In contrast, the red fluorescence increased to 72.6% after AA treatment compared with METH. Taken together, these results indicate that AA protects human dopaminergic cells against mitochondrial injury caused by METH.

## Discussion

Methamphetamine (METH) can act upon the neurons as a central processor of inflammation by releasing pro-inflammatory molecules [7]. These pro-inflammatory molecules may further activate downstream apoptotic signaling pathways in neurons, ultimately resulting in neuronal death and/or the activation of glial cells, which can further exacerbate neuroinflammation [2]. Therefore, inhibition of neurotoxicity and neuroinflammation by METH is important. Pro-inflammatory cytokines TNFα and IL-6 are key mediators of inflammatory responses associated with various neurological disorders including Parkinson’s disease, Alzheimer’s disease, and amyotrophic lateral sclerosis [14]. A high dose of METH has been shown to induce TNFα and IL-6 in the striatum and hippocampus [1].

The major findings of this study are as follows: First, asiatic acid (AA) inhibits METH-induced TNFR overexpression and pro-inflammatory cytokine production in the dopaminergic SH-SY5Y cells. Second, METH-induced NF-κB and STAT3 translocation and ERK phosphorylation are prevented by AA pretreatment in dopaminergic SH-SY5Y cells, mesencephalic neurons, and BV2 cells. Third, AA prevents METH-induced neurotoxicity through the mitochondria-dependent apoptotic pathway in dopaminergic SH-SY5Y cells.

TNFα is considered a potent stimulator of IL-6 production, whose pleiotropic action can be triggered through TNFR [9]. METH significantly increased the TNFR expression in dopaminergic SH-SY5Y cells. However, AA attenuated the elevation of METH-mediated TNFR expression in a concentration dependent.

METH has been found to be associated with neurotoxicity mediated by increased expression of pro-inflammatory cytokines such as TNFα and IL-6 [14]. These cytokines have also been suggested to play an important role in METH-induced brain dysfunction [1]. Our result showed that AA inhibits METH-induced secretion of TNFα and IL-6 and their mRNA expression in SH-SY5Y cells, mesencephalic neurons, and BV2 cells.

METH is able to induce pro-inflammatory cytokines and mediators through the NF-κB and STAT3 pathways in dopaminergic and microglia cells [9, 15, 39]. The potential involvement of NF-κB and STAT3 was investigated because the promoters of both TNFα and IL-6 contain binding sites for NF-κB and STAT3, which are known to be involved in neurological disorders associated with increased inflammation [14, 18]. In accordance with these findings, our results showed that AA effectively inhibits nuclear translocation of NF-κB (p65) and STAT3 in METH-stimulated dopaminergic SH-SY5Y cells, mesencephalic neurons, and BV2 cells. Thus, AA is likely to be a potent inhibitor of NF-κB and STAT3 signaling.

Besides, MAPK signaling pathways play key roles in the induction of inflammatory cytokines by METH [40]. METH has been shown to affect phosphor-ERK and p38 MAPK [41] and monocyte-derived dendritic cells [42]. ERK is a kinase that plays a role in regulating neuronal and behavioral processes mediated by dopamine and glutamate pathways [43]. Our results indicated that AA strongly inhibits METH-induced phosphorylation of ERK. However, AA did not participate in the inhibition of phosphorylation of p38.

Several lines of evidence suggest an important role of the intracellular signal transduction pathways in the mechanism of neural plasticity in response to drug abuse [44]. One of these signal transduction pathways is the ERK1/2 cascade, a member of the mitogen-activated protein kinase (MAPK) family. ERK1/2 activation can phosphorylate tyrosine hydroxylase (TH) and stimulate dopamine synthesis in the brain [45]. Accordingly, we found that phosphor-ERK was most pronounced among all MAPKs after METH treatment. AA completely suppressed TH and phospho-ERK expression in TH-positive mesencephalic neurons. These results suggest that AA regulated TH expression by the inhibition of ERK by METH.

METH-induced cell death contributes to the pathogenesis of neurotoxicity [46, 47]. Previous studies have suggested that apoptosis is a critical phenomenon involved in METH-induced neurotoxicity [11]. Putative mechanisms of METH-induced neurotoxicity have been proposed, including oxidative stress, excitotoxicity, and mitochondrial dysfunction followed by activation of caspase-3 with subsequent cleavage of PARP and DNA fragmentation, appearance of apoptotic cells, and dopaminergic degeneration [3, 48]. We evaluated the effect of AA on METH-induced neurotoxicity in dopaminergic SH-SY5Y cells to gain a better understanding of the molecular mechanisms involved in AA’s anti-apoptotic effects. AA protected dopaminergic SH-SY5Y cells from apoptosis, as evaluated by assays of cell cycle arrest and mitochondrial membrane potential. Our results demonstrated that METH-induced cleavage of PARP was decreased through the inhibition of caspase-3 cleavage by AA. In addition, Bcl-xL was upregulated, whereas Bax was downregulated by AA in METH-induced neurotoxicity. The protective effects became more pronounced when the dopaminergic SH-SY5Y cells were treated with AA.

## Conclusions

Our findings show that AA has a protective role against METH-induced neuroinflammation and neurotoxicity, specifically through inhibition of the NF-kB/STAT3/ERK and mitochondria-mediated apoptosis pathway. Based on the literatures and our findings, AA is a promising candidate for anti-neurotoxic agent, and it can be used for the prevention and treatment of various neurological disorders.

## Additional files

Additional file 1: Figure S1. Effects of AA and METH on the viability and morphology of SH-SY5Y cells. (a) SH-SY5Y cells were treated with AA (1, 5, 10, 15, 20, and 30 μM) for 24 h, and then, MTT assays were conducted (*n* = 4). (b) SH-SY5Y cells were treated with METH (0.5, 1, 1.5, 2, and 5 mM) and then, MTT (*n* = 4) and ELISA assays (*n* = 4) were conducted. (c) SH-SY5Y cells were pretreated with AA (1, 10, and 20 μM) for 1 h and then stimulated with 1 mM METH for 24 h. AA significantly increased the viability of 1 mM METH-stimulated SH-SY5Y cells in a concentration dependent (*n* = 4). (d) Cell morphology changes (magnifications ×200, *n* = 4/group). The data are representative of three independent experiments and quantified as mean values ± SEM. Tukey’s multiple comparison test, *p < 0.05 compared to normal control, ^†^ *p* < 0.05 compared to METH treatment. (PDF 197 kb)

Additional file 2: Figure S2. Effects of AA on METH-induced translocation of NF-κB and STAT3 in SH-SY5Y cells. Immunofluorescence double staining for p-NF-κB p65 (green) and p-STAT3 (red) localization. Cells were counterstained with Hoechst 33342 (blue). Magnifications ×200. The data are representative of three independent experiments. (PDF 83 kb)

Additional file 3: Figure S3. Effects of AA on METH-induced translocation of NF-κB and TH expression in mesencephalic neurons. Immunofluorescence double staining for TH (green) and p-NF-κB p65 (red) localization. Cells were counterstained with Hoechst 33342 (blue). Magnifications ×200. The data are representative of three independent experiments. (PDF 154 kb)

Additional file 4: Figure S4. Effects of AA on METH-induced translocation of STAT3 and TH expression in mesencephalic neurons. Immunofluorescence double staining for TH (green) and p-STAT3 (red) localization. Cells were counterstained with Hoechst 33342 (blue). Magnifications ×200. The data are representative of three independent experiments. (PDF 159 kb)

Additional file 5: Figure S5. METH induced neurotoxicity in dopaminergic SH-SY5Y cells. (a) The cell morphology by phase contrast to understand METH’s neurotoxic effect. (b) Cleaved caspase-3 and PARP were increased with METH treatment. β-actin was used to confirm equal sample loading. The data are representative of three independent experiments and quantified as mean values ± SEM. Tukey’s multiple comparison test, *p < 0.05 compared to normal control. (PDF 174 kb)

## Abbreviations

AA: Asiatic acid
ERK: Extracellular signal-regulated kinase
IL: Interleukin
MAPK: Mitogen-activated protein kinase
METH: Methamphetamine
NF-κB: Nuclear factor kappa-light-chain-enhancer of activated B cells
PD: Parkinson’s disease
STAT: Signal transducer and activator of transcription
TH: Tyrosine hydroxylase
TNF: Tumor necrosis factor
TNFR: Tumor necrosis factor receptor
