# Supplementary figures and images for: Asiatic acid attenuates methamphetamine-induced neuroinflammation and neurotoxicity through blocking of NF-kB/STAT3/ERK and mitochondria-mediated apoptosis pathway
Source: J Neuroinflammation. 2017 Dec 11;14:240. doi: 10.1186/s12974-017-1009-0 (PMC5725763; doi:10.1186/s12974-017-1009-0)

(a)

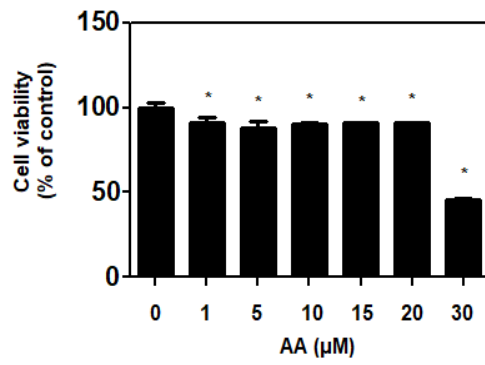

(c)

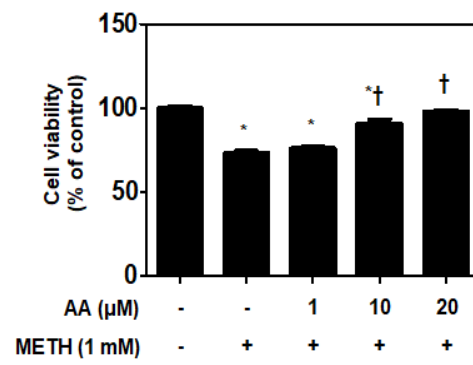

(b)

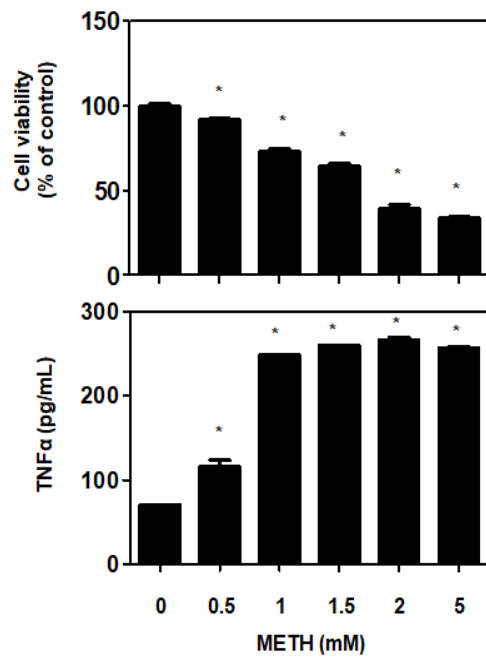

(d)

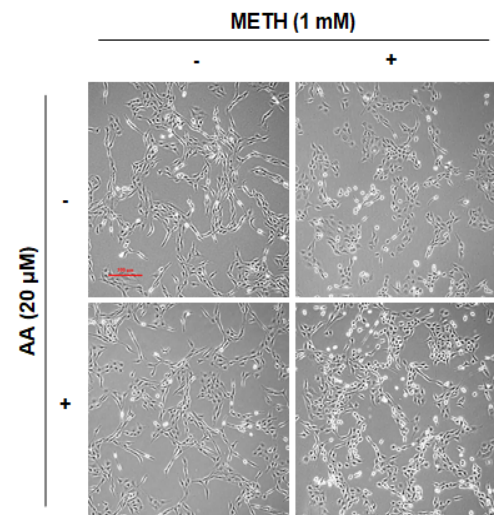

Supplement: Supplementary file 1 — Effects of AA and METH on the viability and morphology of SH-SY5Y cells. (a) SH-SY5Y cells were treated with AA (1, 5, 10, 15, 20, and 30 μM) for 24 h, and then, MTT assays were conducted (n = 4). (b) SH-SY5Y cells were treated with METH (0.5, 1, 1.5, 2, and 5 mM) and then, MTT (n = 4) and ELISA assays (n = 4) were conducted. (c) SH-SY5Y cells were pretreated with AA (1, 10, and 20 μM) for 1 h and then stimulated with 1 mM METH for 24 h. AA significantly increased the viability of 1 mM METH-stimulated SH-SY5Y cells in a concentration dependent (n = 4). (d) Cell morphology changes (magnifications ×200, n = 4/group). The data are representative of three independent experiments and quantified as mean values ± SEM. Tukey’s multiple comparison test, *p < 0.05 compared to normal control, † p < 0.05 compared to METH treatment. (PDF 197 kb) [file 12974_2017_1009_MOESM1_ESM.pdf]

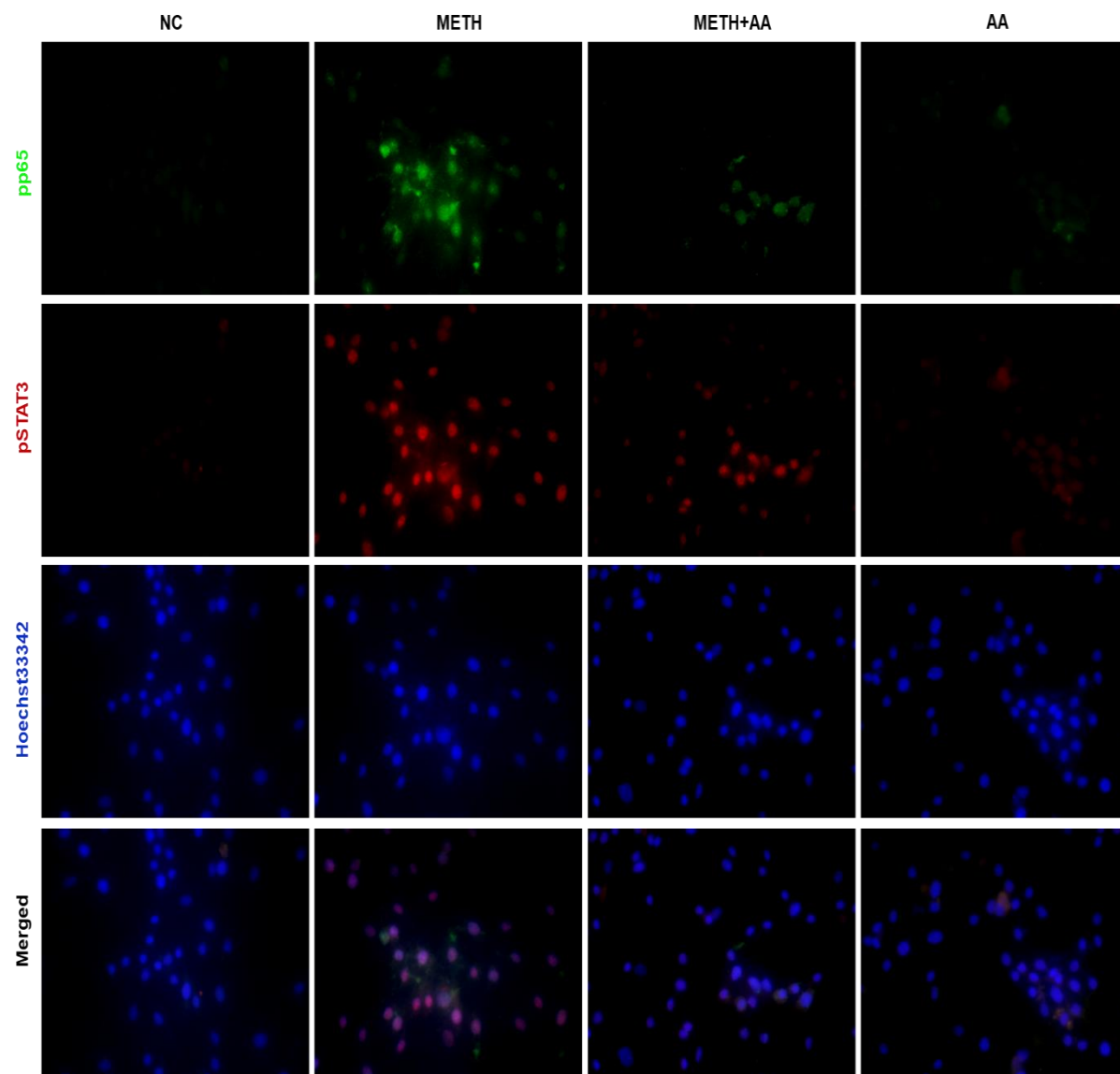

Supplement: Supplementary file 2 — Effects of AA on METH-induced translocation of NF-κB and STAT3 in SH-SY5Y cells. Immunofluorescence double staining for p-NF-κB p65 (green) and p-STAT3 (red) localization. Cells were counterstained with Hoechst 33342 (blue). Magnifications ×200. The data are representative of three independent experiments. (PDF 83 kb) [file 12974_2017_1009_MOESM2_ESM.pdf]

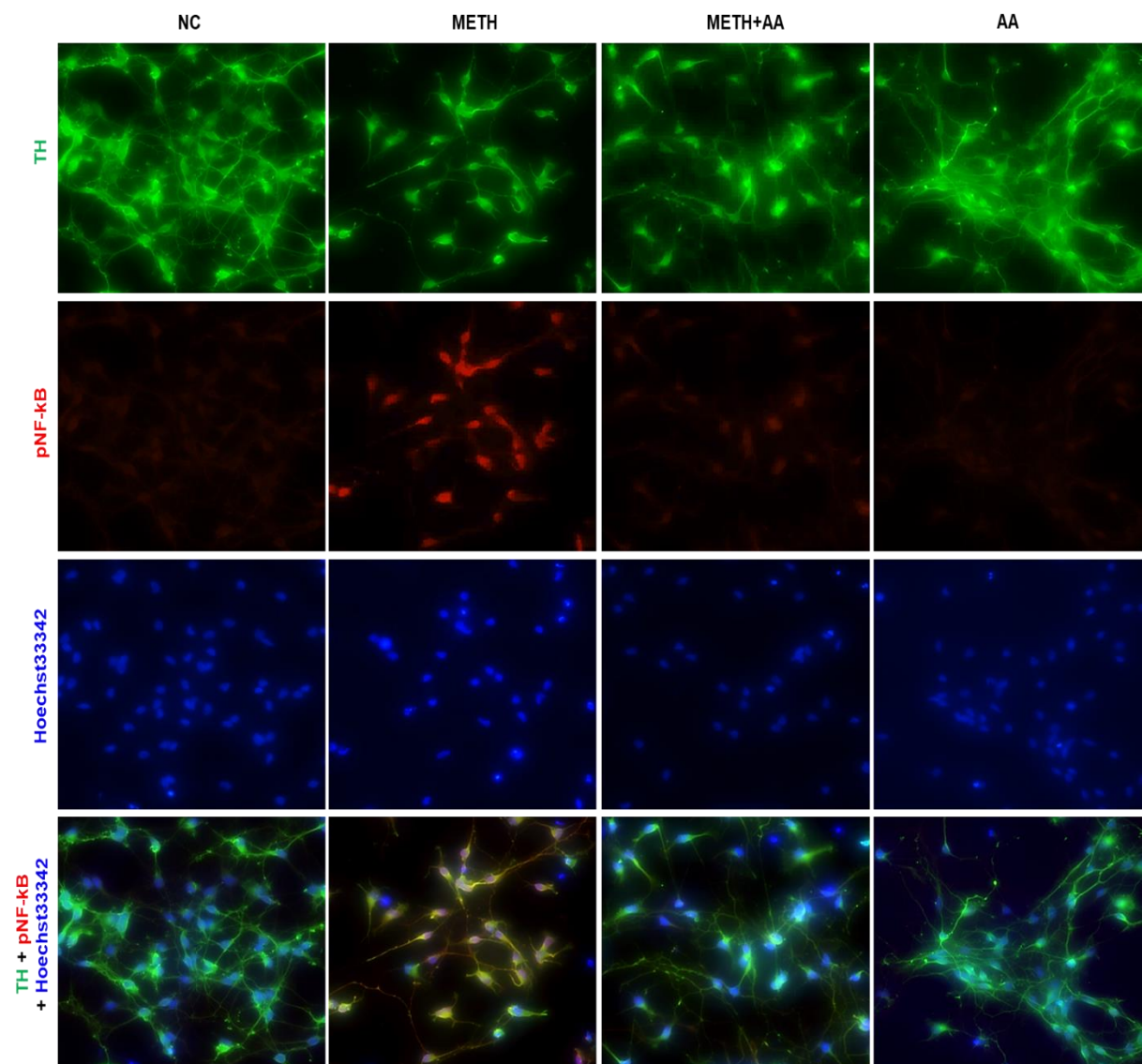

Supplement: Supplementary file 3 — Effects of AA on METH-induced translocation of NF-κB and TH expression in mesencephalic neurons. Immunofluorescence double staining for TH (green) and p-NF-κB p65 (red) localization. Cells were counterstained with Hoechst 33342 (blue). Magnifications ×200. The data are representative of three independent experiments. (PDF 154 kb) [file 12974_2017_1009_MOESM3_ESM.pdf]

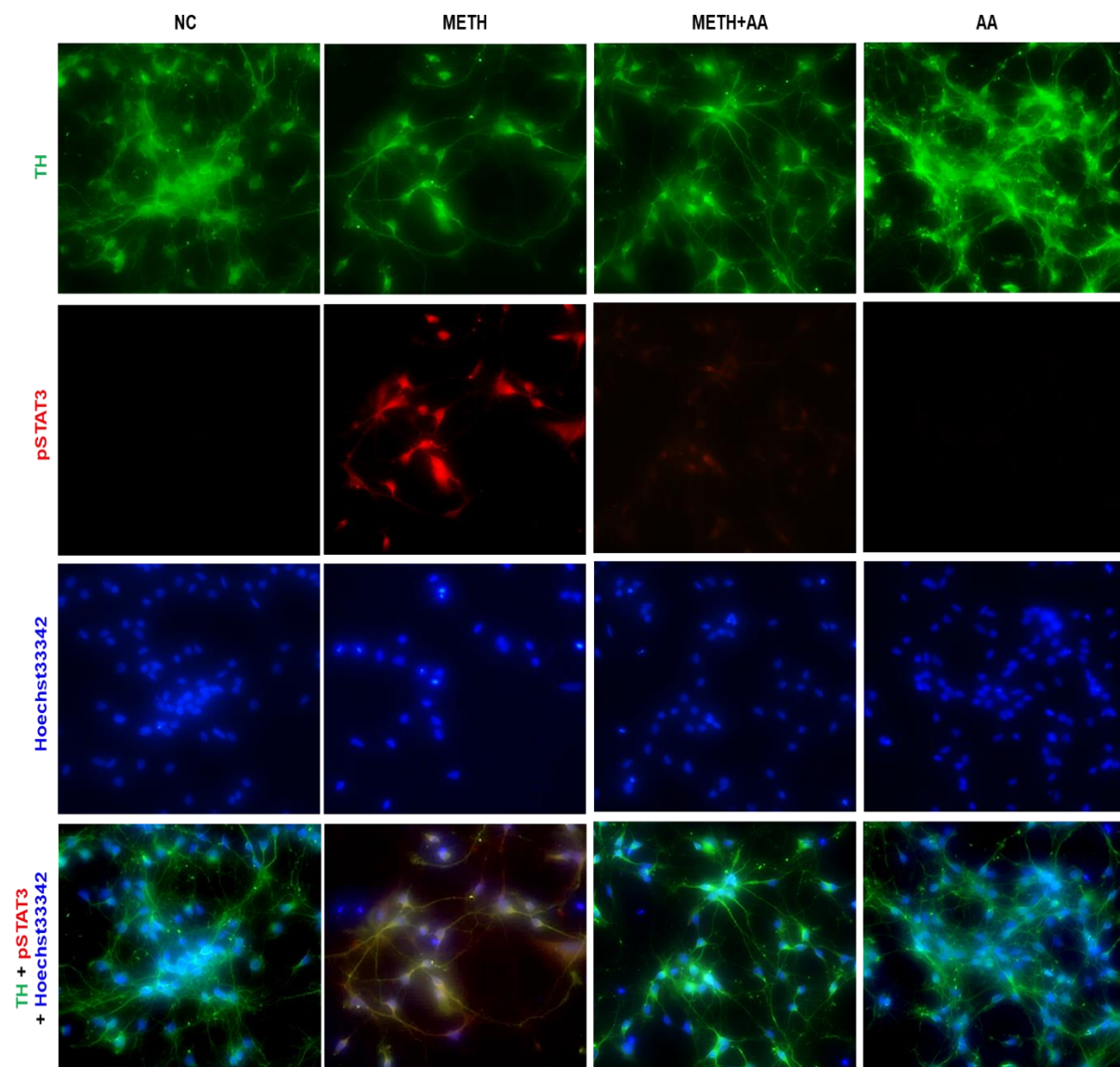

Supplement: Supplementary file 4 — Effects of AA on METH-induced translocation of STAT3 and TH expression in mesencephalic neurons. Immunofluorescence double staining for TH (green) and p-STAT3 (red) localization. Cells were counterstained with Hoechst 33342 (blue). Magnifications ×200. The data are representative of three independent experiments. (PDF 159 kb) [file 12974_2017_1009_MOESM4_ESM.pdf]

(a)

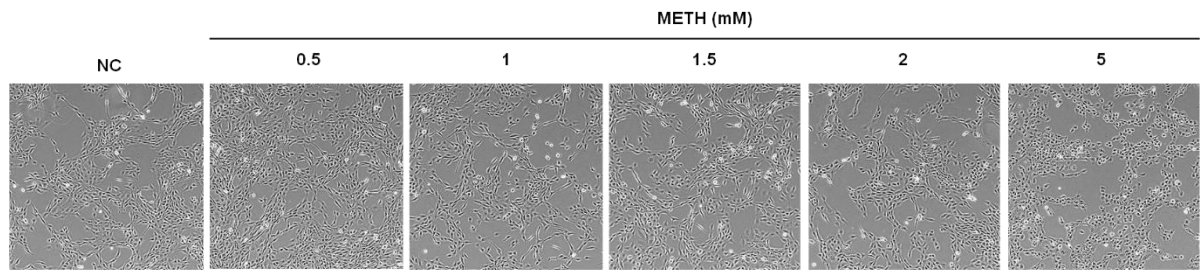

(b)

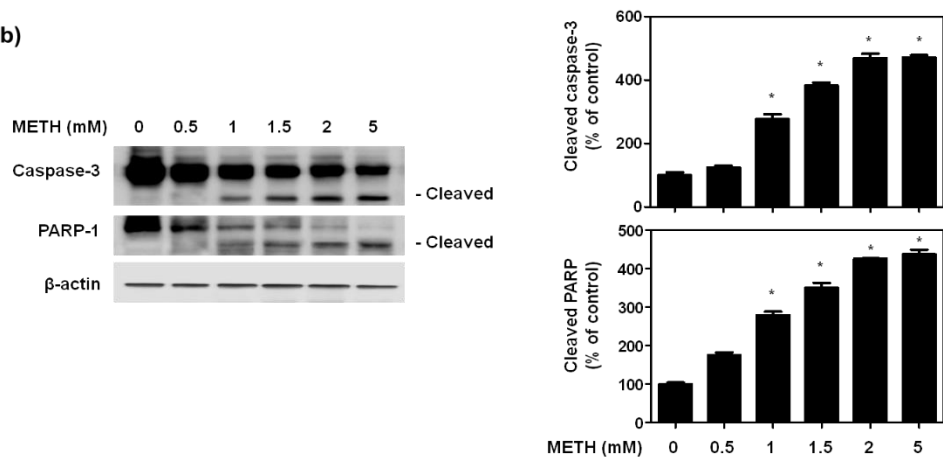

Supplement: Supplementary file 5 — METH induced neurotoxicity in dopaminergic SH-SY5Y cells. (a) The cell morphology by phase contrast to understand METH’s neurotoxic effect. (b) Cleaved caspase-3 and PARP were increased with METH treatment. β-actin was used to confirm equal sample loading. The data are representative of three independent experiments and quantified as mean values ± SEM. Tukey’s multiple comparison test, *p < 0.05 compared to normal control. (PDF 174 kb) [file 12974_2017_1009_MOESM5_ESM.pdf]
